# Computerized tomography scan in acute appendicitis with eventual negative appendectomy

**Published:** 2021-05-27

**Authors:** Ming Li Chia, Kwan Justin, Hui Terrence Chi Hong, G. Shelat Vishal

**Affiliations:** ^1^Lee Kong Chian School of Medicine, Singapore; ^2^Department of Radiology, Tan Tock Seng Hospital, Singapore; ^3^Department of General Surgery, Tan Tock Seng Hospital, Singapore

**Keywords:** negative appendectomy, CT scan, acute appendicitis

## Abstract

**Background and Aim:**

Acute appendicitis (AA) is traditionally considered a clinical diagnosis and negative appendectomy (NA) rates vary across health-care systems. Computed tomography (CT) scans have been shown to aid in the reduction of NA rates. Our study aimed to determine the pre-operative imaging characteristics in patients undergoing appendectomy with eventual normal histology.

**Materials and Methods:**

An audit of all patients with a discharge diagnosis of AA was conducted from January 2011 to December 2015. Histology reports of all patients who underwent appendectomies were reviewed, and medical records of patients with NA were included in the study. To study the impact of CT scan reporting in NA patients, CT scan images of patients with NA were reviewed retrospectively by two blinded radiologists.

**Results:**

A total of 2603 patients underwent appendectomy for suspected AA, and NA rate was 3.34% (n=87). The mean age of patients with NA was 30.3 (14.8-69.8) years with no gender difference (51.7% male). Sixty-six (75.9%) patients had laparoscopic appendectomy with 3.5% open conversion rate. CT scans were done in 47 patients. Pre-operative CT scan report was more likely to report dilated appendix (n=26 [55.3%] vs. n=7 [14.9%], P=0.0001). Post-operative blinded radiology review was more like to report other pathology (n=27 [57.4%] vs. n=2 [4.3%], P=0.0001) and normal appendix (n=26 [55.3%] vs. n=5 (10.6%), P=0.0001).

**Conclusion:**

The NA rate is low. There needs to be standardized reporting for imaging features of prominent/dilated appendix.

**Relevance for Patients:**

Appendectomy must be avoided in patients with a normal CT scan and when another pathological diagnosis is established. Liberal imaging policy assists to reduce NA rates. Imaging features of prominent or dilated appendix can be subjective and international collaboration is needed to define thresholds for imaging diagnosis of AA.

## 1. Introduction

Acute appendicitis (AA) is one of the most common acute surgical conditions. AA’s lifetime risk is 8.6% in males and 6.9% in females, and the appendectomy rate is 12% and 23%, respectively [1,2]. An estimated 50,000 and 300,000 appendicectomies are performed annually in the U.K. and the U.S., respectively [3]. Without appendectomy, complications such as perforation and sepsis-driven organ failure occur. Perforation is a function of late presentation, delay in diagnosis, or prolonged observation and occurs in up to 13.9-16.5% of patients with AA [4,5].

AA is traditionally considered a clinical diagnosis. Abdominal pain is a common reason to seek emergency medical attention, and AA being a common diagnosis, it is not uncommon to diagnose AA based on clinical profile. Hence, clinical diagnosis reliance leads to overdiagnosis and treatment with eventual negative appendectomy (NA) [6]. NA is variably defined as an absence of inflammation or pathology in the appendix or absence of intramural neutrophils in the appendix after an appendectomy [7]. NA is usually a result of over-reliance on clinical judgment, unavailability, or reluctance to obtain imaging investigations, observing a dictum that “appendicitis is a clinical diagnosis” and low operative threshold in young adults with right iliac fossa symptoms. NA rates vary across health-care systems from 6.4 to 30.6% [5,8-10].

Removal of a normal appendix is associated with morbidity, which is more than diagnostic laparoscopy alone and comparable to removing an inflamed appendix [3]. Morbidities include superficial surgical site infection, ileus, urinary retention, urinary tract infection, hematuria, post-operative myocardial infarction, antibiotics use, and attendant morbidity, post-operative intra-abdominal fluid collections, skin reaction to wound dressing, and death [5,11]. In an attempt to increase diagnostic accuracy, clinical prediction rules, such as the Alvarado score, Raja Isteri Pengiran Anak Saleha Appendicitis score, World Society of Emergency Surgery sepsis severity score, Andersson’s inflammatory score, and liberal use of abdominal imaging, are proposed [12-15]. Computed tomography (CT) scans have been shown to aid in the reduction of NA rates. According to Jones *et al.*, a CT scan improves diagnostic accuracy and reduces the NA rate to 2% [16]. Rao *et al*. noted that CT scan availability reduced the NA rate from 20% to 7% in all patients and 3% in patients with a positive CT scan [17]. False-positive CT scans can also lead to an increase in AA’s diagnosis with eventual NA [18]. Our study aimed to determine the relevance of pre-operative imaging in patients undergoing appendectomy with eventual normal histology.

## 2. Materials and Methods

We conducted a retrospective medical record review of all patients with a discharge diagnosis of AA from January 2011 to December 2015. The university affiliated medical center has an estimated annual appendectomy caseload of 500 patients. All patients with the right iliac fossa symptoms are admitted to the general surgical unit and managed according to the duty registrar’s clinical judgment. We liberally perform a CT scan for the evaluation of right iliac fossa symptoms [19]. Our hospital does not have pediatric and gynecological services, and thus, abdominal ultrasound and magnetic resonance imaging are rarely done in the context of AA. We offer both open and laparoscopic appendectomies to patients. We use a grid iron or Lanz incision to perform open appendectomy and a three-port technique for laparoscopic appendectomy. All patients with laparoscopic appendectomy were catheterized before surgery. The base is secured with a ligature or clips [11]. Drains are left at the discretion of the surgeon. Patients are gradually progressed from liquid feeds to diet and managed by standard care pathway. We do not routinely continue antibiotics beyond 48-72 h in patients with uncomplicated AA.

For this study, we reviewed histology reports of all patients who underwent an appendectomy for suspected AA. We audited medical records of patients with normal appendix histology. All patients were reviewed in the outpatient clinic at 1 month post-operative, and none of the patients was diagnosed with alternative diagnosis. To achieve the study aim, we compared the clinical profile of patients with and without CT scans. Furthermore, in patients who had a CT scan performed, we compared the CT scan report with a retrospective review by two blinded independent radiologists.

### 2.1. Pre-operative CT scan

CT scan (if performed) images of patients with NA were reviewed retrospectively by two independent radiologists. Both the radiologists were blinded to the final histology outcome at the time of imaging review. For objective reporting, we provided both radiologists with a standard reporting questionnaire. The questionnaire included 10 predetermined variables and an option to make additional comments. The 10 predetermined variables were generated from existing reports. The phrases and terms commonly used by reporting radiology in our hospital were retained to avoid any reporting bias. Thus, ambiguous terms such as “mildly prominent ‘appendix,’ as well as non-descriptor term ‘appendicitis’” were retained. The ten predetermined variables include that of the normal appendix, mural enhancement and thickening, periappendiceal fat stranding, mildly prominent appendix, dilated appendix (defined as >6 mm diameter on CT scan), mesenteric lymphadenopathy, appendicitis, fluid collection, appendix not seen, and any other incidental pathology noted. The response was recorded as “yes” or “no” with the option for any qualitative comments as deemed fit. In discordance cases, a consensus was reached by internal discussions and mutual agreement between the two radiologists. For this study, we compiled the unified opinion of the two independent radiologists for direct comparison of a unified post-operative opinion with pre-operative imaging. The senior author evaluated the comments. We compared this unified opinion with the original CT scan reporting.

### 2.2. Data collection

We recorded demographic profile, clinical presentation, serum inflammatory markers, and pre-operative CT scan findings. We evaluated each NA patient for age, gender, clinical symptoms, history of diabetes mellitus, vital signs at presentation, clinical signs on abdominal examination, laboratory test results, Alvarado score, mental state, and presence of systemic inflammatory response syndrome (SIRS). To study the outcomes of NA, we looked at access, operative time, insertion of a surgical drain, length of hospital stays, superficial surgical site infection, and intra-abdominal fluid collection.

### 2.3. Definitions

NA is variably defined in the literature as the absence of inflammation or pathology in the appendix or absence of intramural neutrophils in the appendix [7]. For our study, we defined NA as “absence of inflammation or pathology in the appendix” because the histology reports at our hospital do not routinely mention the presence of neutrophils. The absence of reporting cannot rule out AA. We define operative time as time from skin incision to the application of wound dressing. The conversion was defined as a change to open surgery after a laparoscopic attempt. In patients with symptoms or signs of sepsis, a full septic workup includes blood culture, urine culture, chest radiograph, and pertinent serum biochemistry. All patients with a deviation in the post-operative recovery process were actively managed with the liberal use of CT scans in detecting complications. An intra-abdominal fluid collection was diagnosed when a CT scan shows any amount of loculated fluid in the peritoneal cavity, regardless of its size and extent. We calculated the hospital length of stay from the date of surgery to the date of discharge.

### 2.4. Statistical analysis

We summarized patient characteristics and study results using proportions and means or medians with minimum and maximum ranges using the Microsoft Excel software. We also used the independent samples test to compare continuous variables between groups. *P*<0.05 was considered statistically significant. We reported age in mean and all other observations as median (Table 1).

**Table 1:** Clinical profile of patients with normal appendix

	Total n=87(%)	CT scan done (n=47) (%)	CT scan not done (n=40) (%)	*P*-value
Mean age, years (range)	30.3 (14.8-69.8)	32.6 (14.8-69.8)	27.6 (16.2-45.1)	0.0441
Female	42 (48.3)	34 (72.3)	8 (20)	<0.001
Anorexia	22 (25.3)	10 (21.3)	12 (30)	0.351
Nausea or vomiting	40 (46)	28 (59.6)	12 (30)	0.006
Right lower abdominal pain	76 (87.4)	40 (85.1)	36 (90)	0.494
Right lower abdominal tenderness	79 (90.8)	41 (87.2)	38 (95)	0.212
Rebound tenderness	22 (25.3)	6 (12.8)	16 (40)	0.004
Generalized rigidity/guarding	19 (21.8)	8 (17.0)	11 (27.5)	0.238
Temperature (°C) (median) (range)	37.2 (36-39.7)	37.0 (36.2-39)	37.5 (36-39.7)	0.051
>38°C or <36°C	10 (11.5)	2 (4.3)	8 (20.0)	0.022
Heart rate (beats/minute) (median) (range)	88 (60-123)	88 (60-123)	91 (60-120)	0.356
Heart rate >90/min	38 (43.7)	17 (36.2)	21 (52.5)	0.126
Respiratory rate (median) (range)	17 (14-20)	17 (14-20)	17(15-20)	0.859
≥2 SIRS criteria	33 (37.9)	13 (27.7)	20 (50)	0.338
WBC count (H×10^9^/L) (median) (range)	12.5 (2.6-21.9)	10.8 (2.6-21.9)	13.8 (7.6-18.9)	0.016
Systolic blood pressure (mm of Hg) (median) (range)	119 (95-165)	118 (97-157)	120.5 (95-165)	0.289
Diastolic blood pressure (mm of Hg) (median) (range)	69 (44-109)	68 (51-109)	69 (44-96)	0.533
Neutrophilia (%) (median) (range)	74.9 (48.9-94)	68.2 (48.9-94)	78.1 (63.6-92.9)	0.002

CT: Computerized tomography, SIRS: Systemic inflammatory response syndrome, WBC: White blood cell

## 3. Results

Our hospital performed 2594 appendicectomies between January 2011 and December 2015. Eighty-seven patients (3.3%) had an absence of inflammation or pathology in the appendix on histopathology examination. The mean age of 87 patients with NA was 30.3 (14.8-69.8) years with no gender difference (51.7% male). Twenty-two (25.3%) patients experienced anorexia, 40 (46.0%) patients experienced nausea or vomiting, and 76 (87.4%) patients had migratory right lower abdominal pain. Right lower abdominal tenderness, rebound tenderness, and guarding were present in 79 (90.8%), 22 (25.3%), and 19 (21.8%) patients, respectively (Table 1). The median temperature, heart rate, blood pressure, respiratory rate, white blood cell count (WBC), and neutrophil count were 37.2°C, 88/min, 119/69mmHg, 17/min, 12.5×10^9^/L, and 74.9%, respectively (Table 1). None of the 87 patients had altered mental state. Alvarado score suggested probable to the very probable diagnosis of AA in 36 (41.4%) patients, and 33 (37.9%) patients met SIRS criteria (Table 1). Sixty-six (75.9%) patients had laparoscopic appendectomy with 3.5% open conversion rate. The median operative time was 75 (35-190) min, and the median length of hospital stay was 2.1 (0.9-14.7) days. Table 2 shows the perioperative outcomes of patients with eventual NA on histology. Seven (8.0%) patients developed superficial surgical site infections. One patient (1.1%) developed an intra-abdominal abscess, and there was nil mortality.

**Table 2:** Scoring systems and operative outcomes of patients with normal appendix

	Total n=87 (%)	CT scans done (n=47)	CT scans not done (n=40)	*P*-value
Alvarado score (median) (range)	6 (2-10)	6 (2-9)	6 (2-10)	0.096
5-6 (compatible)	34 (39.1)	17 (36.2)	17 (42.5)	-
7-8 (probable)	31 (35.6)	16 (34.0)	15 (37.5)	-
9-10 (very probable)	5 (5.7)	2 (4.3)	3 (7.5)	-
Glasgow coma score 15	87 (100)	47 (100)	40 (100)	-
Laparoscopic approach	66 (75.9)	39 (83.0)	27 (67.5)	0.016
Converted to open	3 (3.4)	3 (6.4)	0 (0)	
Operative time (median) (minutes) (range)	75 (35-190)	75 (35-160)	70 (35-190)	0.264
Median hospital stay (median) (days) (range)	2.0 (0.8-14.6)	2.4 (0.8-14.6)	1.8 (1.0-12.1)	0.053
Surgical drain inserted	3 (3.4)	3 (6.4)	Nil	-
Superficial surgical site infection	7 (8.0)	6 (12.8)	1 (2.5)	0.118
Intra-abdominal abscess	1 (1.1)	Nil	1 (2.5)	-
Mortality	Nil	Nil	Nil	-

CT: Computerized tomography

### 3.1. Pre-operative CT scan report and post-operative CT scan review

**Figure 1:**
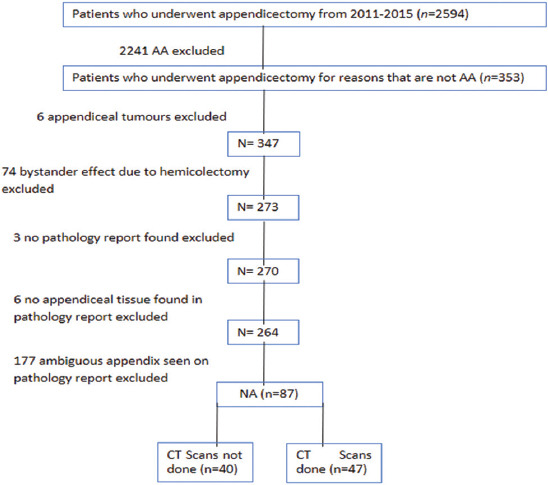
Histology results of appendicectomies and selection of patients with normal appendix

**Figure 2:**
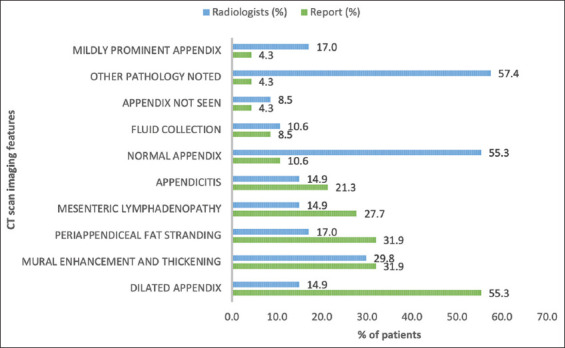
Comparison of CT scan findings between preoperative reporting and postoperative review. The preoperative CT scan report was associated with dilated appendix (*P*<0.0001), and postoperative CT scan image review was associated with a normal appendix, and other pathology noted (*P*<0.0001 for both). There was no significant difference in other variables.

Of these 87 patients, CT scans were done in 47 patients (Figure 1). Figure 2 shows the reported CT scan and independent radiologist CT scan review findings of 47 patients who had a CT scan performed. We observed several discordances between the pre-operative CT scan report and the post-operative CT scan review by two blinded independent radiologists. Pre-operative CT scan report was more likely to report dilated appendix (n=26 [55.3%] vs. n=7 [14.9%], P=0.0001). Post-operative blinded independent radiology review was more like to report other pathology (n=27 [57.4%] vs. n=2 [4.3%], P=0.0001) and normal appendix (n=26 [55.3%] vs. n=5 [10.6%], *P*=0.0001). Pre-operative report and post-operative review did not show statistically significant difference for mural enhancement and thickening (31.9% vs. 29.8%, *P*=0.823), periappendiceal fat stranding (31.9% vs. 17%, *P*=0.149), mesenteric lymphadenopathy (27.7% vs. 14.9%, *P*=0.208), appendicitis (21.3% vs. 14.9%, *P*=0.592), fluid collection (8.5% vs. 10.6%, *P*=0.726), appendix not visualized (4.3% vs. 8.5%, *P*=0.677), and mildly prominent appendix (4.3% vs. 17%, *P*=0.09), respectively.

### 3.2. Patients with CT scan and without CT scan

Comparing the 47 patients who underwent a CT scan with the 40 patients who did not undergo a CT scan, there was a significant difference in an age where patients with CT scans done were older (32.6 [14.8-69.8] vs. 27.6 [16.2-45.1] years, *P*=0.044), and there were more female patients (34 [72.3%] vs. 8 [20%], *P*<0.001) who had a CT scan. Further, patients who experienced nausea or vomiting were more likely to be scanned (59.6% vs. 30%, *P*=0.006). More patients in the group without CT scans had pyrexia >38°C or <36°C (20% vs. 4.3%, *P*=0.022). Heart rate, respiratory rate, and blood pressure were similar between the two groups. Overall, in the group without CT scans, more patients had SIRS criteria, but this was not significant (50.0% vs. 27.7%, *P*=0.338). Patients without a CT scan were more likely to have rebound tenderness (40.0% vs. 12.8%, *P*=0.004), higher median white blood cell count (13.8 H×10^9^/L vs. 10.8 H×10^9^/L, *P*=0.016), and neutrophilia (78.1% vs. 68.2%, *P*=0.002). Alvarado score was similar between both groups. Patients without a CT scan were more likely to receive an open appendectomy (32.5% vs. 17%, *P*=0.016). Median operative time and hospital stay were similar between the two groups. More patients in the CT scan group developed short-term complications with regard to a surgical drain (6.4% vs. nil) and superficial surgical site infection (12.8% vs. 2.5%, *P*=0.118), but this was not significant. One patient (2.5%) in the group who did not undergo a CT scan group developed an intra-abdominal abscess. We did not encounter pneumonia, deep vein thrombosis, myocardial infarction, cerebrovascular accident, urinary tract infection, appendix stump blowout, or iatrogenic bowel injuries in our study.

## 4. Discussion

In patients operated for suspected AA with eventual normal histology, temperature >38°C or <36°C, rebound tenderness, elevated total white blood cell count, and neutrophilia are associated with clinical decision for appendectomy. Further, older patients, females, and patients with nausea or vomiting were more likely to receive a CT scan, and this is also within expectations to rule out other possible etiology for right lower iliac fossa symptoms. Independent blinded radiology assessors reported “normal appendix” and “other incidental pathology” more often when compared to the preoperative CT scan report.

NA rate is low in our experience (3.3%) compared to other studies [10,20,21]. According to Jones *et al.*, the appropriate CT scan utilization to aid in diagnosing AA should decrease the NA rate to 2% [16]. Rao *et al*. also noted that the availability of a CT scan coincided with a reduction in the NA rate from 20% to 7% in all patients, and they observed 3% false-positive CT scan [16,17]. Our institution adopts a liberal use of CT scans, and still, almost half of the patients did not receive a CT scan. CT scan order for patients with the right iliac fossa symptoms is based on individual judgment, and non-ordering is multifactorial. Radiation risk associated with CT scan is real, and hence, clinical judgment is prudent in young patients [22]. According to Malik *et al.*, using CT scans to diagnose AA has a slight benefit above and beyond the traditional blood and clinical tests [23]. In addition, CT scan does not alter the endpoint of AA [23]. They suggested that scraping the CT scans for possible appendicitis could save the health service 4.3 million pounds in 2014 throughout the U.K., and the percentage of NA remains similar to that in the absence of investigations [23]. However, our study has shown that CT scans significantly reduce the rate of NA. Reducing the rate of NA is essential as the removal of a histologically normal appendix appears to carry more morbidity than a diagnostic laparoscopy alone and is comparable with that of removing an inflamed appendix [24]. In our experience, patients who had CT scans are more likely to have increased length of stay compared to upfront appendectomy patients. However, this was not significant due to the small sample size. This is likely due to the waiting interval between admissions to CT scan and then delay in the decision for surgery, that is, the increase in the length of stay is not due to operative morbidity. More patients with upfront appendectomy had an open approach, and this could be due to adverse clinical signs and inflammation markers.

Appendectomy should be avoided in patients with a normal CT scan, especially when another clinical diagnosis is established. From our study, patients who did not undergo a CT scan tend to have a higher degree of systemic inflammation as well as more prominent abdominal signs, and thus, upfront surgery was offered. An abdominal CT scan for suspected appendicitis has sensitivity and specificity rates between 76-100% and 83-100%, respectively [25]. In our institution, despite the liberal use of CT imaging in cases of suspected AA, there are still cases of NA. Due to false-positive CT scan reporting for AA, adopting a universal policy of CT scan also would not be a cost-effective strategy, and in our opinion, there is a low baseline rate of NA, which is challenging to eliminate.

Radiology reports commonly use phrases such as “mildly prominent appendix” and “dilated appendix >6 mm” to suggest a diagnosis of AA. Ultrasound size criteria for appendicitis of >6 mm are not applicable for CT scan as normal appendix can measure >6 mm on CT scan [26]. Furthermore, the diameter of a normal appendix on a CT scan ranges from 6 to 10 mm, with 42% of appendices larger than 6 mm, possibly due to intraluminal content [27]. According to Charoensak *et al.*, in a retrospective review of CT scans of 538 patients without clinical suspicion of AA, the mean outer diameter of the appendix was 6.6 mm ± 1.5 and mean wall thickness of the appendix was 4.4 mm ± 1.0 [28]. Therefore, to further reduce the rates of NA, CT scan reporting terms such as periappendiceal fat stranding, prominent, or dilated appendix need standardization.

To clarify this aspect, we conducted a blinded CT image review by two independent radiologists asking for structured reporting along with qualitative feedback. In 172 patients with equivocal CT scan in AA patients, Daly *et al*. have recruited two radiologists to reassess appendiceal size, presence of the right lower quadrant stranding, fluid, or an appendicolith [29]. Authors reported that 119 (69%) of 172 patients with equivocal findings on CT scans did not have AA. Similarly, we found a disparity between blinded radiology reporting versus the original CT scan report (Figure 2). CT scan reporting in an acute setting may be more likely to report periappendiceal stranding to exclude the diagnosis of AA by the duty surgical team. The independent blinded radiology opinion reporting half of the patients as having a normal appendix is interesting and is likely due to blinding. The independent review also reported higher rates of “mildly prominent appendix,” which could have been interpreted by the emergency duty radiologist as “dilated appendix.” This is likely related to reporting bias, given the clinical history of suspected AA. Hence, there is a need to standardize the terms “dilated appendix” and “mildly prominent appendix” to increase the objectiveness of CT scan reporting. Literature has also shown that between 6 and 10 mm diameter, there is an overlap between a normal appendix and AA on CT scan [26]. In our study, we have defined dilated appendix as a maximum outer wall to outer wall diameter of >6 mm. This finding is very sensitive (97.5%) but less specific (59.6%), hence the term “non-specific,” especially when there are confounding factors such as fluid within the appendiceal lumen. Conversely, a max diameter of >8.2 mm is less sensitive (88.8%) but more specific (93.4%) [28]. Hence, in clinical practice, 6-8 mm appendix is labeled as indeterminate and more than 8 mm as suggestive of AA.

Our study has several limitations. This study is a single-center retrospective audit of patients with a normal appendix on histology, and the significance of clinical signs and inflammation markers must be considered as an association and not causation. However, it is unlikely that a prospective randomized study will be deemed ethical on this theme, and hence, these data are important. We did not routinely do C-reactive protein, serum procalcitonin, or serum albumin in patients presenting with the right iliac fossa symptoms and hence unable to report all the inflammatory markers. We did not collect data from patients with histology-proven appendicitis, and unable to comment on the true-positive and false-negative rate of CT scan. In patients with clinical features suspicious of AA but normal CT scan, clinician judgment should take precedence and management guided by local protocols, resources, as well as experience. We retrospectively calculated the Alvarado score, and hence, our study has reporting bias. Finally, the two blinded independent radiologists would also have known that the project was something related to “appendix” as they were tasked to report on ten specific findings related to AA. Hence, this could introduce information bias in their opinion. In addition, we combined the report of both radiologists to formulate a unified report. Thus, we were unable to report inter-rater reliability among the two radiologists.

## 5. Conclusion

In conclusion, appendectomy should be avoided in patients with a normal CT scan and when another pathological diagnosis is established. Despite liberal policy to CT scan, there are still instances of NA, and this baseline rate of NA is challenging to eliminate. There needs to be standardized reporting by duty radiologists for imaging features of prominent/dilated appendix.
